# Mobitz Type II Atrioventricular Block Followed by Remifentanil in a Patient with Severe Aortic Stenosis

**DOI:** 10.1155/2013/852143

**Published:** 2013-04-03

**Authors:** Mehryar Taghavi Gilani, Majid Razavi

**Affiliations:** Anesthesia Department, Imam-Reza Hospital, School of Medicine, Mashhad University of Medical Sciences, Mashhad, Iran

## Abstract

Opioids have been considered for their hemodynamic stability. Remifentanil is an opioid analgesic with rapid metabolism and fast primary effect and recovery. In this paper, a very rare effect of using remifentanil along with propofol was presented. An 84-year-old male patient with severe aortic stenosis underwent general anesthesia. In order to induce anesthesia and maintain it, fentanyl, pancuronium, and propofol, along with a combination of propofol and remifentanil, were used, respectively. At beginning of remifentanil infusion, bradycardia and then Mobitz type II conduction block with a hemodynamic disorder occurred for the patient. The decreased blood pressure responded to injection of atropine and ephedrine; however, dysrhythmia only improved after cessation of remifentanil. Therefore remifentanil should be used with caution in aortic stenosis.

## 1. Introduction

Remifentanil is a congener of fentanyl family of narcotics which is separable from others for the ester structure. Complications of this drug are like those of other opioids and include bradycardia, itching, nausea, vomiting, and muscular rigidity [1]. Aortic stenosis is the most common valvular disorder of heart which is seen in cardiac rheumatic disease and old ages and is divided to slight, moderate, and severe types based on valvular diameter and transvalvular pressure gradient. Anesthesia in these patients could be accompanied by decreased cardiac output, and cardiopulmonary resuscitation is hardly done at this situation. 

In this study, a serious complication of remifentanil was considered during management of anesthesia in a patient with severe aortic stenosis.

## 2. Case Description

The patient was an 84-year-old man, weighting 72 kg, who was hospitalized for open prostate surgery. In records, the patient was only complaining from exertional dyspnea, had no obvious cardiopulmonary problems, and did not mention using any specific drugs. In the conducted examination, pulmonary auscultation had no problems. Examination of abdomen and organs was normal, and only IV/VI systolic murmur and a thrill were heard in the aortic area. In order to evaluate the patient before the operation, blood tests, electrocardiogram, and echocardiogram were requested. The only positive point in the lab tests was prothrombin time (PT) = 15.6 sec. In the electrocardiogram (ECG), heart rate was 65 beats per minute, and the rhythm was regular; however, left axis deviation and left ventricle hypertrophy were observed. In echo, ejection fraction was 57%, severe hypertrophy of left ventricle, calcified and narrow aortic valve were reported; also, 52 mm Hg was reported for transvalvular aortic gradient; but, no functional and wall motion disorder was seen for left ventricle.

**Figure 1:**
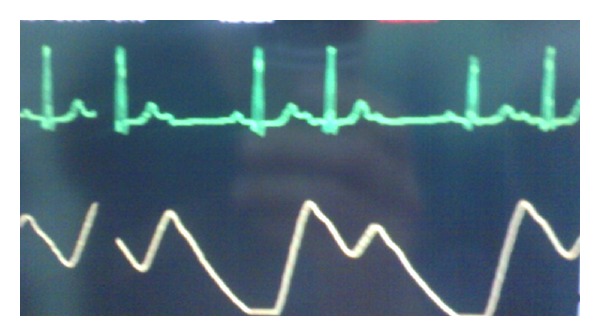
After remifentanil infusion.

**Figure 2:**
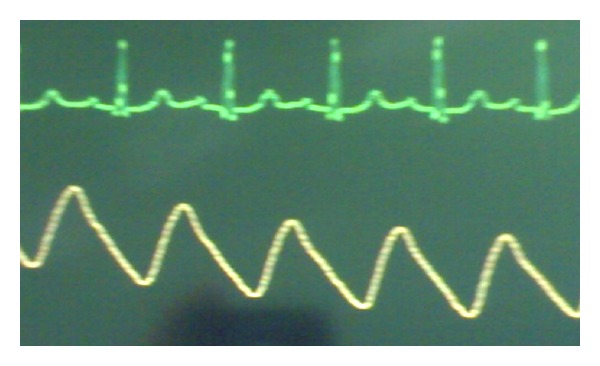
After remifentanil cessation.

Vital signs of the patient before the induction were blood pressure of 150/90 mm Hg and heart rate of 60 beats per minute. For anesthetic induction with caution and low dose, 100 *µ*g of fentanyl, 4 mg of pancuronium, and 80 mg of propofol were used. After tracheal intubation, in order to maintain anesthesia, a combination of propofol and remifentanil (200 mg + 500 *µ*g) along with 50% oxygen and N_2_O was used. The rate of infusion was 0.2 *µ*g/kg/min for remifentanil. After induction and beginning the infusion of remifentanil and propofol, the patient suffered from decreased heart rate (35–40 beats per minute) and decreased blood pressure (80/50 mm Hg); after a few moments, he experienced Mobitz II atrioventricular block (Figure 1). To increase heart rate and sinus control, first, 0.5 mg atropine and then 10 mg ephedrine were injected; however, atrioventricular block was not modified (heart rate of about 40 beats per minute) despite increased blood pressure to 110/75 mm Hg. Then, infusion of remifentanil was stopped and propofol with rate of 50 *µ*g/kg/min was used alone. After about 2-3 min, atrioventricular block and bradyarrhythmia were overcome (Figure 2), and the patient's blood pressure was raised to 130/90 mm Hg. The patient had no hemodynamic problem during the operation, and recovery and was completely under control. He had a little restlessness during the recovery and improved after some minutes. Then, he was transferred to the ward in total awareness with blood pressure of 130/80 and heart rate of 62 beats per minute. 

## 3. Discussion

Remifentanil is a narcotic from the fentanyl's family, which has a rapid primary effect and has a short half life (about 3 min) even after long-term usage; therefore, it is a good choice in total intravenous anesthesia (TIVA) and pain relief in ICU [2]. Due to hemodynamic stability, remifentanil is used in many cardiac diseases such as eisenmenger [3], coarctation of aorta [4], and severe aortic stenosis [5, 6], cardiomyopathies [7]. In the elderly, the pharmacokinetic and pharmacodynamic of remifentanil differ, and there is increased sensitivity of brain to remifentanil. Also, potency of the drug is twice, and it is required to decrease primary dosage of the drug. Central distribution volume and also clearance decrease, and the amount of infusion should be decreased to 1/3 [8, 9]. However, in some cases, this drug could decrease cardiovascular function, and the patient may suffer from bradycardia and hypotension. Sometimes, severe variations are observed in heart rate which has been reported as a result of predominance of parasympathetic over sympathetic tone and is seen in the patients with junctional rhythms and even temporary sinusoidal arrest, which rapidly improves by injection of anticholinergic drug (atropine) [10, 11]. In Fattorini et al.'s study, as a consequence of stimulating sinus node with increased heart rate to less than 140 beats per minute, Wenckebach atrioventricular block was observed in 7 out of 40 patients, which indicated specific increase in refractory period of the atrioventricular node [10]. 

Aortic stenosis is one of the most common valvular disorders among the elderly. This disorder occurs in children as a result of bicuspid valve and rheumatic diseases, and, in the elderly, it happens mostly because of degenerative disorders, calcification, and fibrous aortic valve. Aortic stenosis is divided to three: slight, moderate, and severe grades according to the difference in transvalvular gradient (left ventricle and aorta). In the severe and fatal type, gradient difference is more than 50 mm Hg and diameter of aortic valve is less than 0.7 cm^2^ in this case [6]. 

In patients with severe aortic stenosis, performing anesthesia has many risks, and, even performing CPR and using defibrillator have a low degree of success. In these patients, due to hypertrophy of left ventricle, the amount of intravascular liquid should be maintained at a desirable and upper normal level; therefore, sometimes, central venous pressure and pulmonary artery pressure are monitored. Increased heart rate could cause ischemia of heart muscle as a result of the decrease in the circulation of coronary blood along with increase in oxygen demand; also, bradycardia causes decrease in cardiac output. Systemic vascular resistance should be also maintained at normal level in order to maintain cardiac output. Increased resistance with increased gradient prevents aortic flow, and decreased resistance of vessels also causes decreased peripheral perfusion and ischemia problems. Decreased blood pressure should be immediately modified by alpha agonists like phenylephrine and metaraminol. Atrial contraction in normal people includes 15–20% of cardiac output; but, in people with severe aortic stenosis, about 40% of cardiac output is caused by atrial contraction, and any nonsinusoidal rhythm decreases cardiac output.

In the surgeries on patients with severe aortic stenosis, regional anesthesia [12] and also general anesthesia could be used; but, regional anesthesia causes sympathectomy and can decrease peripheral vascular resistance. Moreover, bradycardia which follows anesthesia can decrease cardiac output. Thus, in this patient who had 52 mm Hg differences in gradient pressure, general anesthesia was performed. For hemodynamic stability, an opioid base using infusion of remifentanil was used. Since opioids are not adequately anesthetic by themselves, low dosage of propofol was used for induction, and also remifentanil was applied for infusion. In order to prevent decreased heart rate, pancuronium was used. In this patient, severe decrease of heart rate was accompanied by Mobitz cardiac block type II, and this variation in heart rate and rhythm caused hemodynamic disorder which did not respond to injection of atropine and ephedrine; only stopping remifentanil caused improvement.

There have been a limited number of reports on creation of complete heart block following concurrent consumption of propofol, remifentanil, vecuronium, and sevoflurane [13, 14], but, no case of Mobitz cardiac block type II has been observed. Also, in most of the studies, using anticholinergic before remifentanil or after the rhythm disorder improves or prevents that signs; however, in this patient, using atropine and also indirect sympathomimetic compound of ephedrine did not solve the rhythm disorder. In the study by Mizuno et al. on a 17-year-old patient, an intermittent bundle branch block followed by bradycardia was reported in the consumption of sevoflurane and remifentanil, which responded to atropine [15]. In Nishio et al.'s study, a 66-year-old patient with sick sinus syndrome, propofol was used along with sevoflurane and remifentanil, which did not intensify the disorder in the patient [16]. In Fujii et al.'s investigation (2011), effect of remifentanil on sinus node and atrial conduction was studied among 60 children, in both of them conduction pathway was inhibited, and no disorder was observed in atrioventricular conduction [17], but, in this paper, the patient suffered from atrioventricular conduction disorder and Mobitz type II block followed by bradycardia.

## 4. Final Conclusion

In patients with severe aortic stenosis, remifentanil can be used for hemodynamic stabilization, but, remifentanil along with propofol could cause conduction disorder and conduction block and should be used with caution in aortic stenosis. In this patient, dysrhythmia and atrioventricular block did not respond to atropine and ephedrine treatment, and only remifentanil cessation solved the problem.
